# Single‐cell RNA sequencing reveals the landscapes of human cord blood hematopoietic stem cell differentiation during ex vivo culture

**DOI:** 10.1002/ctm2.616

**Published:** 2021-11-08

**Authors:** Ruiting Wen, Chen Xu, Chen Dong, Hongxia Sheng, Long Zhao, Yang Yang, Ang Zhang, Shenyu Wang, Li Wang, Yan Ju, Yang Liu, Lian Duan, Liangding Hu, Hu Chen, Zhigang Yang, Bin Zhang

**Affiliations:** ^1^ Department of Hematology The Fifth Medical Center of Chinese PLA General Hospital Beijing PR China; ^2^ Department of Hematology Central People's Hospital of Zhanjiang Zhanjiang PR China; ^3^ Academy of Military Medical Sciences Academy of Military Sciences Beijing PR China; ^4^ Department of Obstetrical The Fifth Medical Center of Chinese PLA General Hospital Beijing PR China; ^5^ Department of Neurosurgery The Fifth Medical Center of Chinese PLA General Hospital Beijing PR China

Dear Editor,

Previous findings indicate that SR1, UM171 and a JNK inhibitor JNK‐IN‐8 (K1) can promote HSC expansion.^1^, ^2^, ^3^ However, effect of the ex vivo expansion system on HSC self‐renewal and differentiation is still inadequately understood. Here, we established an expansion strategy using a combination of cytokines (SCF, TPO, FLT3‐L and IL‐6) and USK (UM171, SR1 and K1) that contributed to a 543‐fold increase relative to the initial cell population in CD34^+^CD38^–^CD45RA^–^CD90^+^ cells (Figure 1A–C, Tables S1 and S2). Moreover, colony‐forming units assay confirmed that the cells cultured with USK, UM171 or K1 retain the ability to differentiate into multilineage progenitors (Figure 1D, Table S3). These findings seemed to be very encouraging; however, subsequent findings were unexpected. In primary transplantation, the proportion of engrafted hCD45^+^ cells in the USK group was not as good as that in the uncultured. Only when 500 initial cells were transplanted, there was no difference in the proportion of hCD45^+^ cells between the USK group and the uncultured. However, when 2500 or 10000 initial cells were transplanted, the proportion of hCD45^+^ cells in the USK group and vehicle group was lower than that of the uncultured (Figure 2A–D, Table S4). Additionally, there was no significant difference between the USK group and the uncultured in the frequency of NOD‐SCID repopulating cells (SRCs) (Figure 2E,F, Table S5). These results suggest that the short‐term engraftment ability of uncultured CB CD34^+^ cells is superior to that of cells expanded by USK and vehicle when the number of initial CB CD34^+^ cells is adequate. Subsequently, the data from the secondary transplantation showed that there was no difference in the proportion of hCD45^+^ cells and SRC frequency among all groups (Figure 3, Tables S6 and S7). These findings indicate that cell subsets with a CD34^+^CD38^–^CD45RA^–^CD90^+^ phenotype expanded by USK may not have long‐term engraftment ability.

**FIGURE 1:**
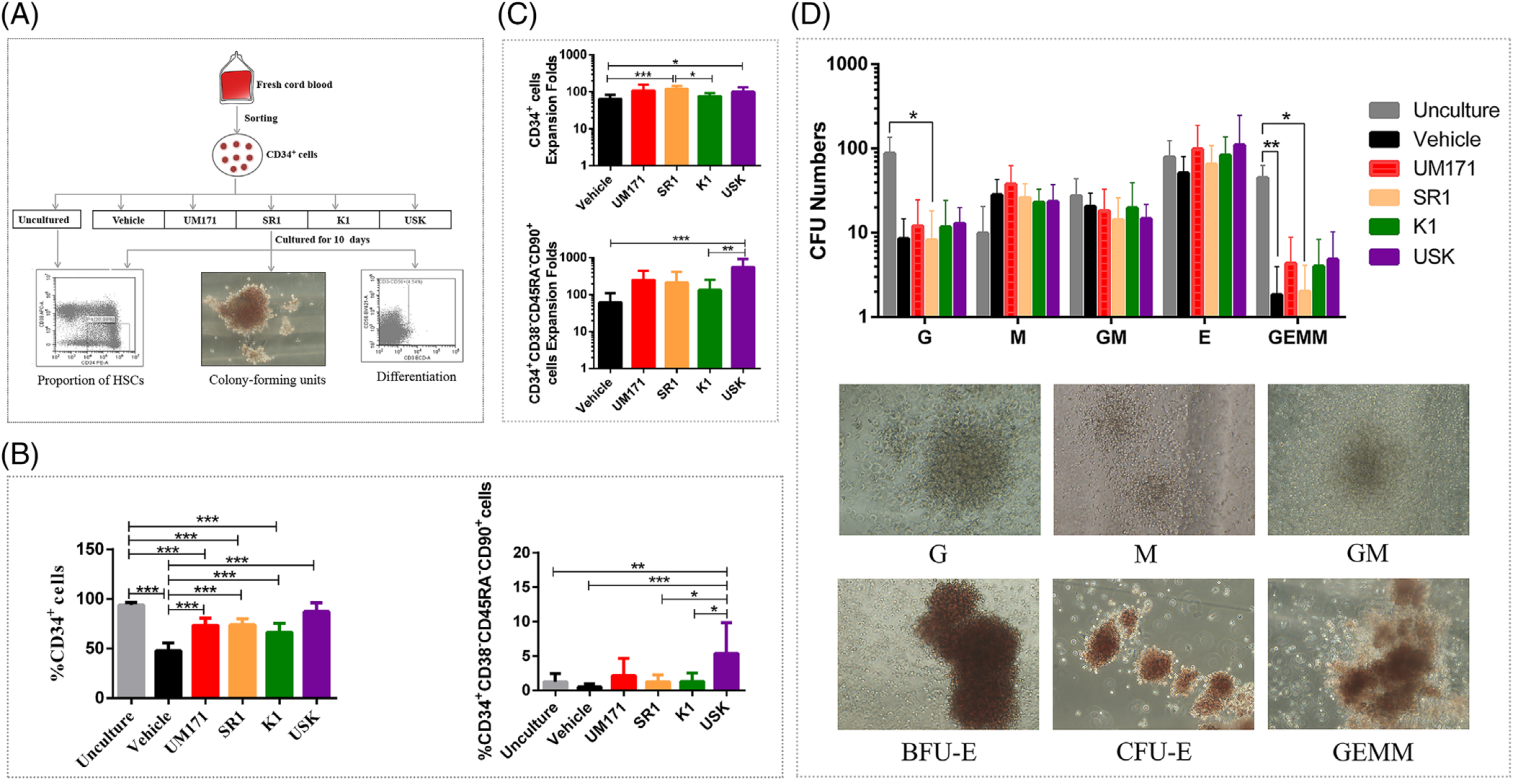
USK promotes the ex vivo expansion of HSCs with the phenotype of CD34^+^CD38^–^CD45RA^–^CD90^+^. (A) Workflow of cord blood derived CD34^+^ cells cultured in vitro. (B) Proportion of CD34^+^ cells and CD34^+^CD38^–^CD45RA^–^CD90^+^ cells in the unculture, vehicle, UM171, SR1, K1 and USK groups (*n* = 11 independent experiments, data shown as mean ± SD, one way ANOVA test). (C) Expansion folds of CD34^+^ cells and CD34^+^CD38^–^CD45RA^–^CD90^+^ cells in the vehicle, UM171, SR1, K1 and USK groups (*n* = 11 independent experiments, data shown as mean ± SD, one way ANOVA test). (D) Uncultured CD34^+^ cells and CB CD34+ cells cultured with vehicle, UM171, SR1, K1, USK for 10 days, which these cells (1 × 10^3^/dish) were separately seeded into cytokine‐containing methylcellulose media and then cultured for 14 days to perform CFU experiment (*n* = 6 independent experiments, data shown as mean ± SD, Kruskal–Wallis test) and representative images of CFU. USK,UM171+SR1+K1; HSCs, hematopoietic stem cells; BFU, burst‐forming units‐erythroid; CFU, colony‐forming units; E, erythrocyte; G, granulocyte; GEMM, granulocyte/ erythrocyte/macrophage/ megakaryocyte; GM, granulocyte‐macrophage; M, macrophage; TNC, total nucleated cells. *p < 0.05; **p < 0.01; ***p < 0.001

**FIGURE 2:**
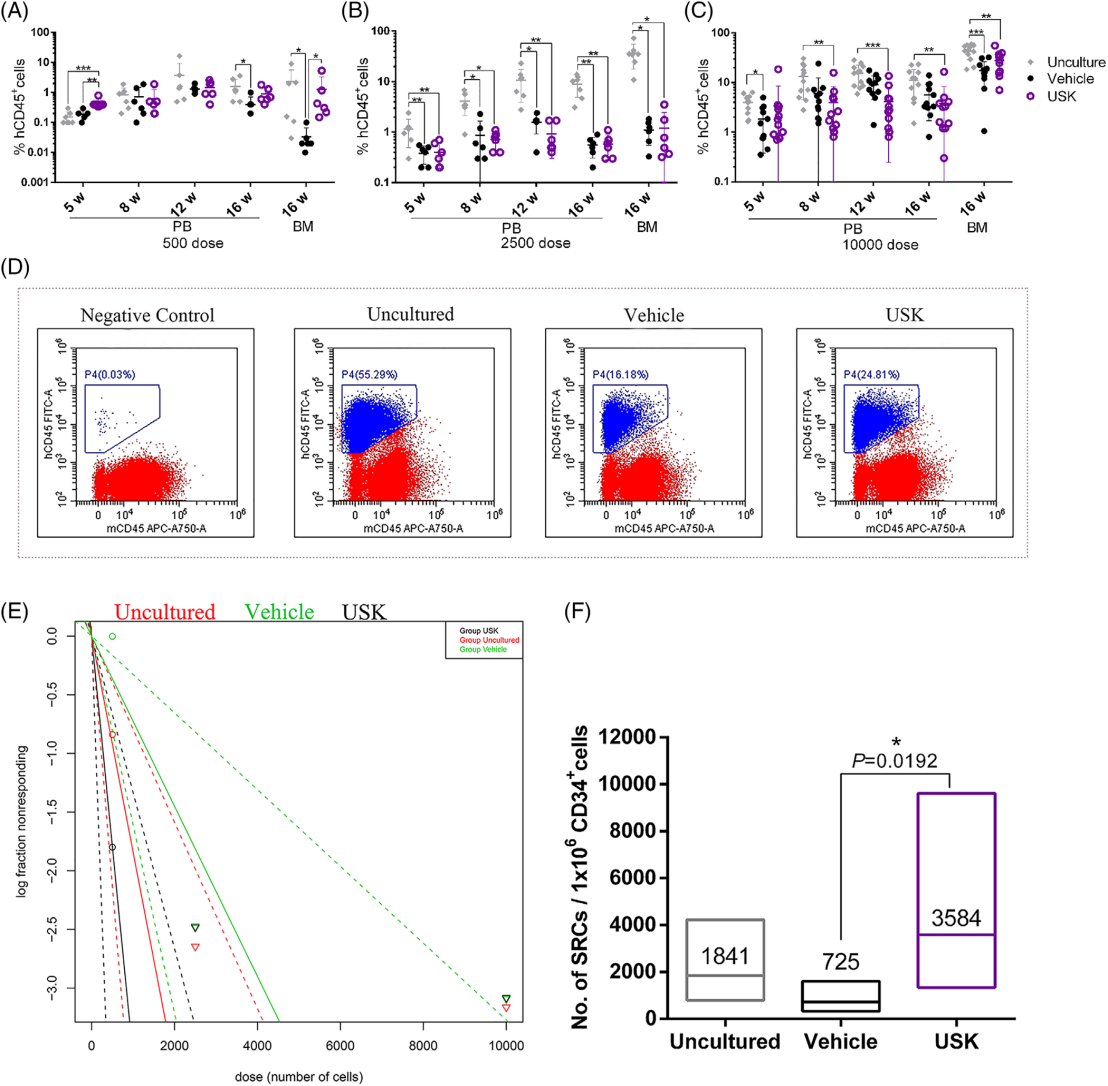
Engraftment of human cells in primary NPG mice. (A) Proportion of hCD45^+^ cells in PB and BM of primary recipients of 500 starting dose (uncultured group *n* = 7, vehicle group *n* = 6, USK group *n* = 6, data shown as mean ± SD, Kruskal–Wallis test). (B) Proportion of hCD45^+^ cells in PB and BM of primary recipients of 2500 starting dose (uncultured group *n* = 7, vehicle group *n* = 6, USK group *n* = 6, data shown as mean ± SD, Kruskal–Wallis test). (C) Proportion of hCD45^+^ cells in PB and BM of primary recipients of 10000 starting dose (uncultured group *n* = 12, vehicle group *n* = 11, USK group *n* = 11, data shown as mean ± SD, Kruskal–Wallis test). (D) Representative flow cytometry plots for hCD45^+^ cell proportion in BM of primary recipients of 10 000 starting dose at 16 weeks post‐transplantation. Non‐transplanted mouse BM cells were used as the negative control. (E) Primary LDA (limiting dilution analysis) to determine the SCID repopulating cell (SRC) frequency by ELDA software at 16 weeks post‐transplantation (data from Table S5A). Dotted lines represent the 95% confidence intervals. Shapes represent the per cent of negative animals for each dose of cells. (F) The number of SRCs in 1×10^6^ CD34^+^ cells was calculated using Poisson statistics applied to the data in Table 5A(data shown as median. Chi‐square test). ELDA, extreme limiting dilution assay; LDA, limiting dilution analysis; NPG, NOD‐Prkdc^scid^ Il2rg^null^; BM, bone marrow; PB, peripheral blood. *p < 0.05; **p < 0.01; ***p < 0.001

**FIGURE 3:**
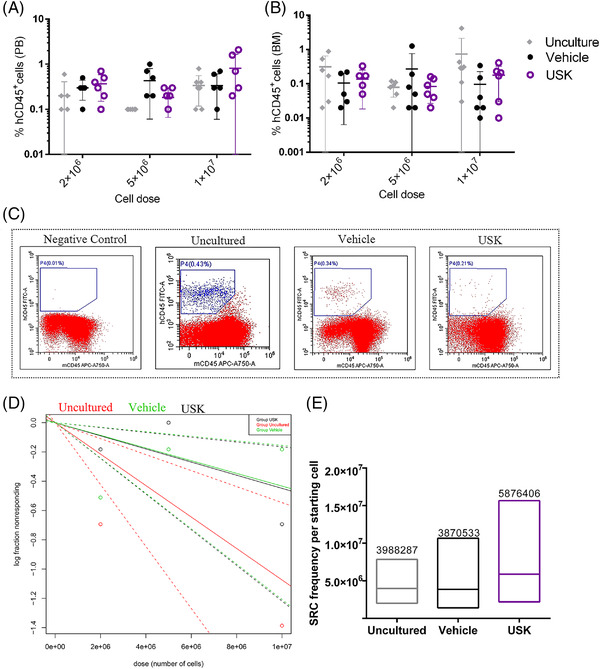
Engraftment of human cells in secondary NPG mice at week 16 post‐transplantation. (A) Proportion of hCD45^+^ cells in PB of secondary recipients (uncultured group 1 × 10^7^ dose *n* = 7, 5 × 10^6^ dose *n* = 6, 2 × 10^6^ dose *n* = 6; vehicle group 1 × 10^7^ dose *n* = 6, 5 × 10^6^ dose *n* = 6, 2×10^6^ dose *n* = 5; USK 1 × 10^7^ dose *n* = 6, 5 × 10^6^ dose *n* = 6, 2 × 10^6^ dose *n* = 6; data shown as mean ± SD, Kruskal–Wallis test). (B) Proportion of hCD45^+^ cells in BM of secondary recipients (uncultured group 1 × 10^7^ dose *n* = 8, 5 × 10^6^ dose *n* = 6, 2 × 10^6^ dose *n* = 6; vehicle group 1 × 10^7^ dose *n* = 6, 5 × 10^6^ dose *n* = 6, 2 × 10^6^ dose *n* = 5; USK 1 × 10^7^ dose *n* = 6, 5 × 10^6^ dose *n* = 6, 2 × 10^6^ dose *n* = 6; data shown as mean ± SD, Kruskal–Wallis test). (C) Representative flow cytometry plots for hCD45^+^ cell proportion in BM of secondary recipients. (D) Limit dilution analysis of secondary recipient engraftment at 16 weeks post‐transplantation (data shown as mean ± SD, Kruskal–Wallis test). The frequencies of SCID repopulating cells (SRCs) were calculated by ELDA software. Data from Table S7A. Dotted lines represent the 95% confidence intervals. Shapes represent the per cent of negative animals for each dose of cells. (E) SRC frequencies per starting cell in secondary recipient(data shown as median. Chi‐square test)

Recent research updated our understanding of HSCs and defined HSCs, myeloid‐restricted stem cells (MySCs) and megakaryocyte‐erythrocyte stem cells (MESCs) as stem cells with bona fide self‐renewal capacity based on their capacity to be transplanted into secondary recipients.^4^ Multipotent‐repopulating progenitors (multiRP) and myeloid‐restricted repopulating progenitors (MyRP), which are reconstituted only in primary recipients, are considered progenitor cells.^4^ To further clarify the effect of the ex vivo expansion system on HSC differentiation, we used single‐cell RNA sequencing (scRNA‐seq) to analyze uncultured CB CD34^+^ cells, CB CD34^+^ cells cultured with cytokines and CB CD34^+^ cells cultured with cytokines plus USK. According to their canonical marker genes, 17 clusters (clusters 0‐16) were annotated (Figure 4A,B, Figure S1 and Table S8). We annotated that C4 cells were HSCs characterized by the HSC‐related transcription factors KLF2, HES1, MLLT3^5^ and MSI2^6^ and the markers AVP,^7^ CD34 and PROM1. C16 was annotated as MySCs in view of cells in this cluster expressing HSC‐related genes and differentiating into myeloid cells.^4^ scRNA‐seq data further confirmed that C4‐HSCs, C16‐MySCs and C10‐MESCs almost disappeared, whereas progenitor cells increased significantly, after CB CD34^+^ cells were cultured with USK for 10 days (Table S9), which was consistent with the expanded cells having no advantage in long‐term engraftment in serial transplantation, indicating that bona fide HSCs in CB CD34^+^ cells had already undergone differentiation during culture.

**FIGURE 4:**
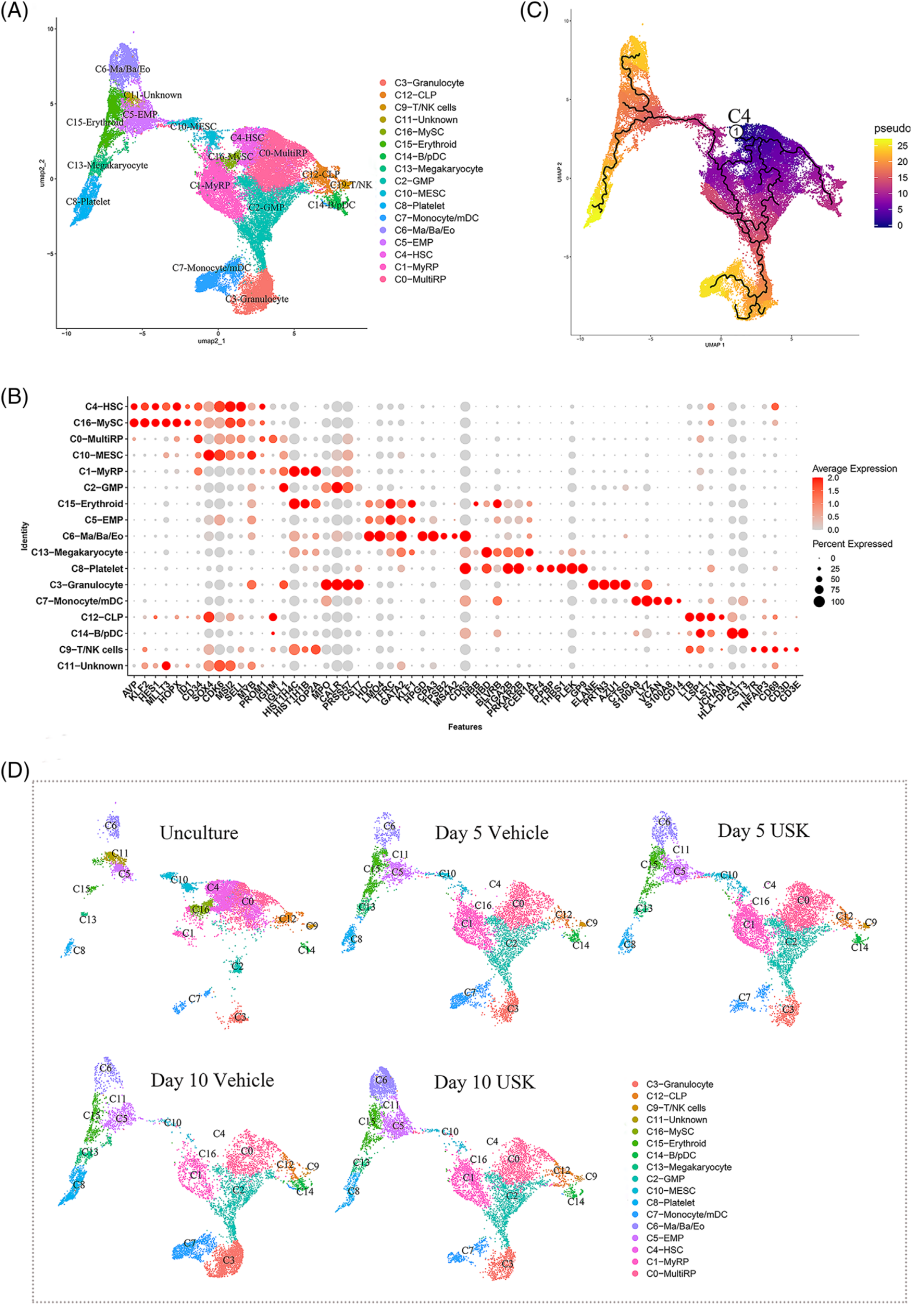
A single‐cell resolution map of CD34^+^cells from cord blood. (A) Uniform manifold approximation and projection (UMAP) clustering map of cord blood CD34^+^ cells from integrated five samples. (B) Dot plot showing representative marker genes of the defined cell clusters. Colour indicates the ratio between the expression level of each group and the maximum expression level of all groups. Size of the dot is proportional to the percentage of cells expressing this gene in each cluster. (C) Trajectory analysis by Monocle 3 reconstructed the path from HSC to multi‐lineage differentiation. (D) UMAP clustering map of CD34^+^ cells under different culture conditions

Recent studies hold that the cell‐surface markers used for isolation of HSCs are inaccurate for ex vivo culture of HSCs.^8^ Similar to our study, Chen et al.^9^ found that ex vivo expanded cells with a phenotype of CD34^+^CD38^–^CD45RA^–^CD90^+^CD49f^+^ were not functional HSCs. Therefore, it may be inappropriate to previously define CD34^+^CD38^–^CD45RA^–^CD90^+^ as a marker for long‐term hematopoietic stem cells (LT‐HSCs) during ex vivo culture. It is important to identify novel markers for supervising the repopulating ability of HSCs. Our results showed that HOPX and ID1 were significantly expressed on C4‐HSCs and C16‐MySCs (Figure 4B). Therefore, we inferred that HOPX and ID1 might be phenotypic markers of human HSCs.

To understand the mechanism underlying the differentiation of HSCs during culture, we compared the differentially expressed genes (DEGs) between USK‐treated and uncultured cells. The results showed that USK treatment up‐regulated the expression of cell proliferation‐related genes and myeloid genes and down‐regulated the expression of the JUN, JUND, FOS, FOSB, IRF1, REL and NFKBIA genes (Figure S2A,B). We further analyzed the DEGs and signaling pathways between C0‐multiRP, C1‐MyRP, C2‐GMP and C4‐HSC. The results showed that the expression of the JUN, JUND, FOS, FOSB, IRF1 and NFKBIA was up‐regulated (Figure S2C), the oxidative phosphorylation pathway was negatively regulated, and the MAPK, FOXO, and REG/GR pathways were positively regulated in C4‐HSCs versus C0‐multiRP, C1‐MyRP and C2‐GMP cells (Figure S3). Additionally, we also compared the DEG between USK and Vehicle treatment. The results showed that USK treatment up‐regulated gene expression of CD34, ALDH1A1, IGLL1, PRDX1 and SELENOW and down‐regulated NEAT1 expression (Figure S4). Finally, we analyzed HSC‐related transcription factors and their target genes. The results showed that FOS, FOSB, JUN, JUND, IRF1 and REL were highly regulated in C4‐HSCs and C16‐MySCs. And cell cycle analysis showed that these transcription factors regulations were in G1 phase (Figure S5). The target genes that interact with these transcription factors included HSC‐related genes, HOX family members, and NF‐κB, TGF‐β, mTOR and MAPK signaling‐related genes. This observation indicates that the disappearance of C4‐HSCs and the increase in progenitor cells may be related to the downregulation of JUN, JUND, FOS, FOSB, IRF1 and NFKBIA, and these transcription factors may be the key molecules regulating HSC self‐renewal.

In conclusion, our findings suggest that USK expanded more than 500‐fold of CD34^+^CD38^–^CD45RA^–^CD90^+^ cells, which can only short‐term engraftment in primary recipients. We for the first time confirmed that previously considered LT‐HSCs with the phenotype of CD34^+^CD38^–^CD45RA^–^CD90^+^ have no self‐renewal ability, and bona fide HSCs have undergone differentiation during ex vivo culture. And scRNA‐seq showed that HOPX and ID1 may be markers of human HSC. FOS, FOSB, JUN, JUND, IRF1 and REL may play an important role in regulating HSC self‐renewal. However, further validation is still needed with more experiments.

## CONFLICT OF INTEREST

The authors declare that they have no competing interests.

## Supporting information

SUPPORTING INFORMATION

SUPPORTING INFORMATION

SUPPORTING INFORMATION

SUPPORTING INFORMATION

SUPPORTING INFORMATION

SUPPORTING INFORMATION

SUPPORTING INFORMATION

SUPPORTING INFORMATION

SUPPORTING INFORMATION

SUPPORTING INFORMATION

SUPPORTING INFORMATION

SUPPORTING INFORMATION

SUPPORTING INFORMATION

SUPPORTING INFORMATION

SUPPORTING INFORMATION

SUPPORTING INFORMATION
