# Supplementary figures and images for: Single‐cell RNA sequencing reveals the landscapes of human cord blood hematopoietic stem cell differentiation during ex vivo culture
Source: Clin Transl Med. 2021 Nov 8;11(11):e616. doi: 10.1002/ctm2.616 (PMC8574970; doi:10.1002/ctm2.616)

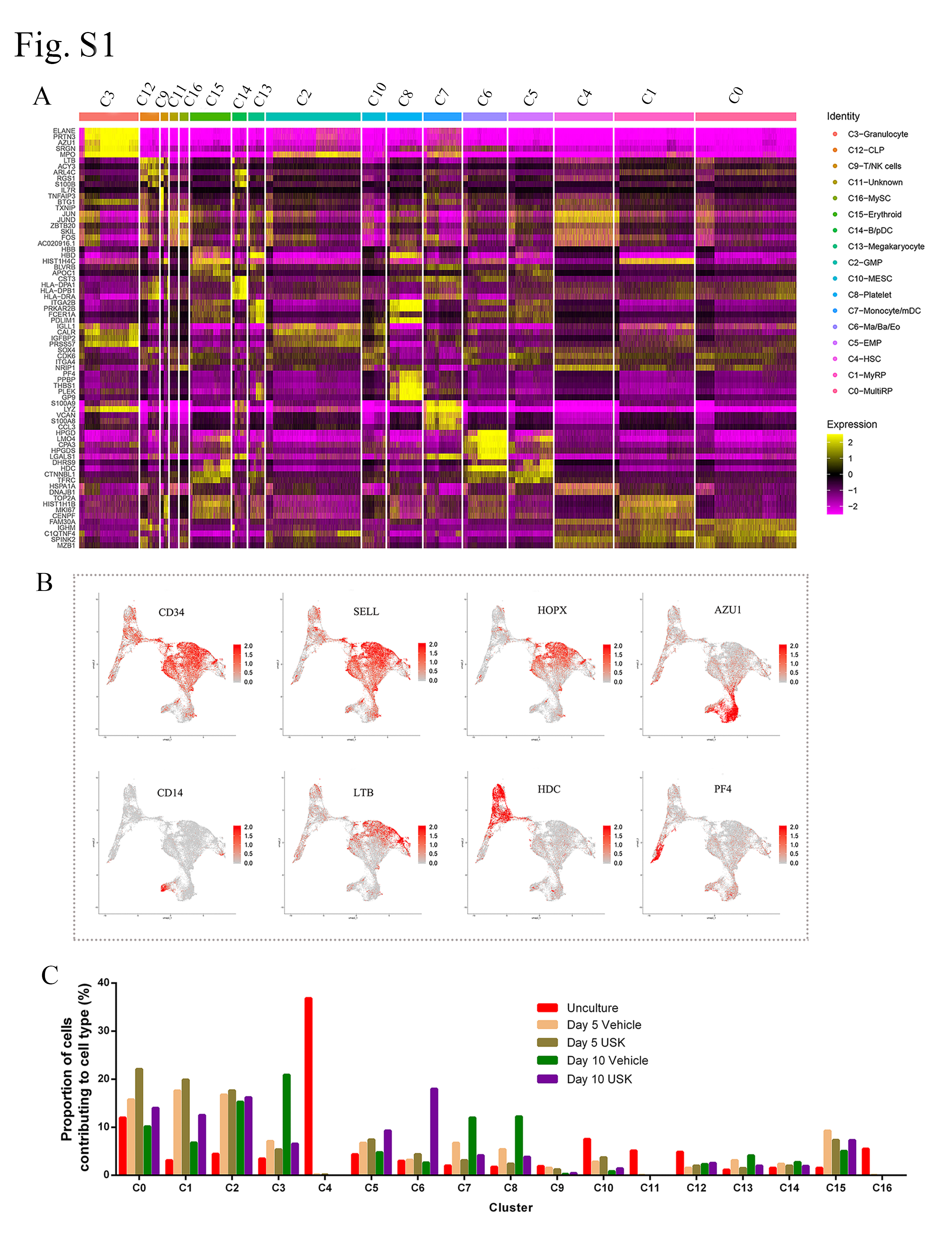

Supplement: Supplementary file 1 — SUPPORTING INFORMATION [file CTM2-11-e616-s003.tif]

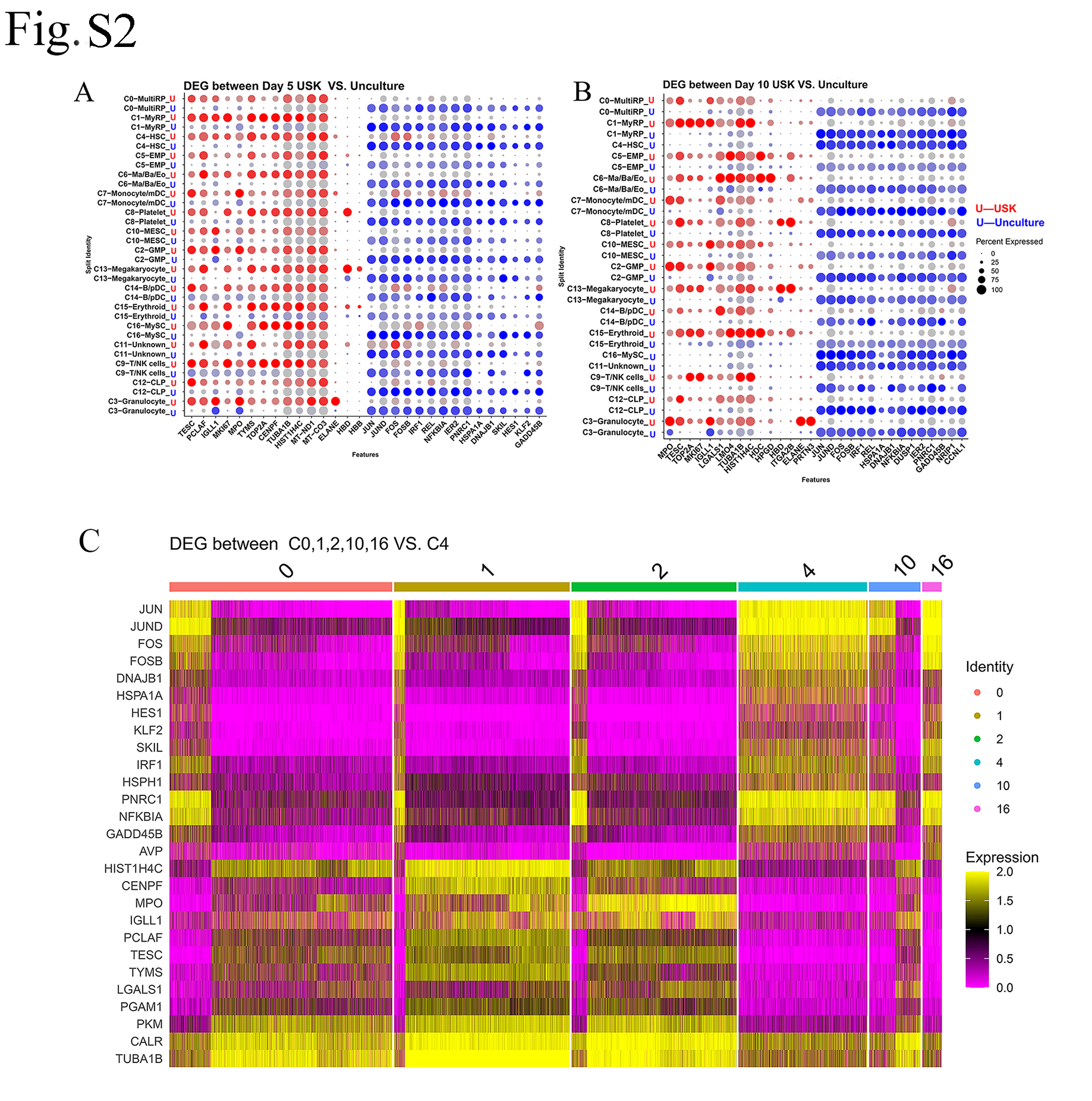

Supplement: Supplementary file 2 — SUPPORTING INFORMATION [file CTM2-11-e616-s005.tif]

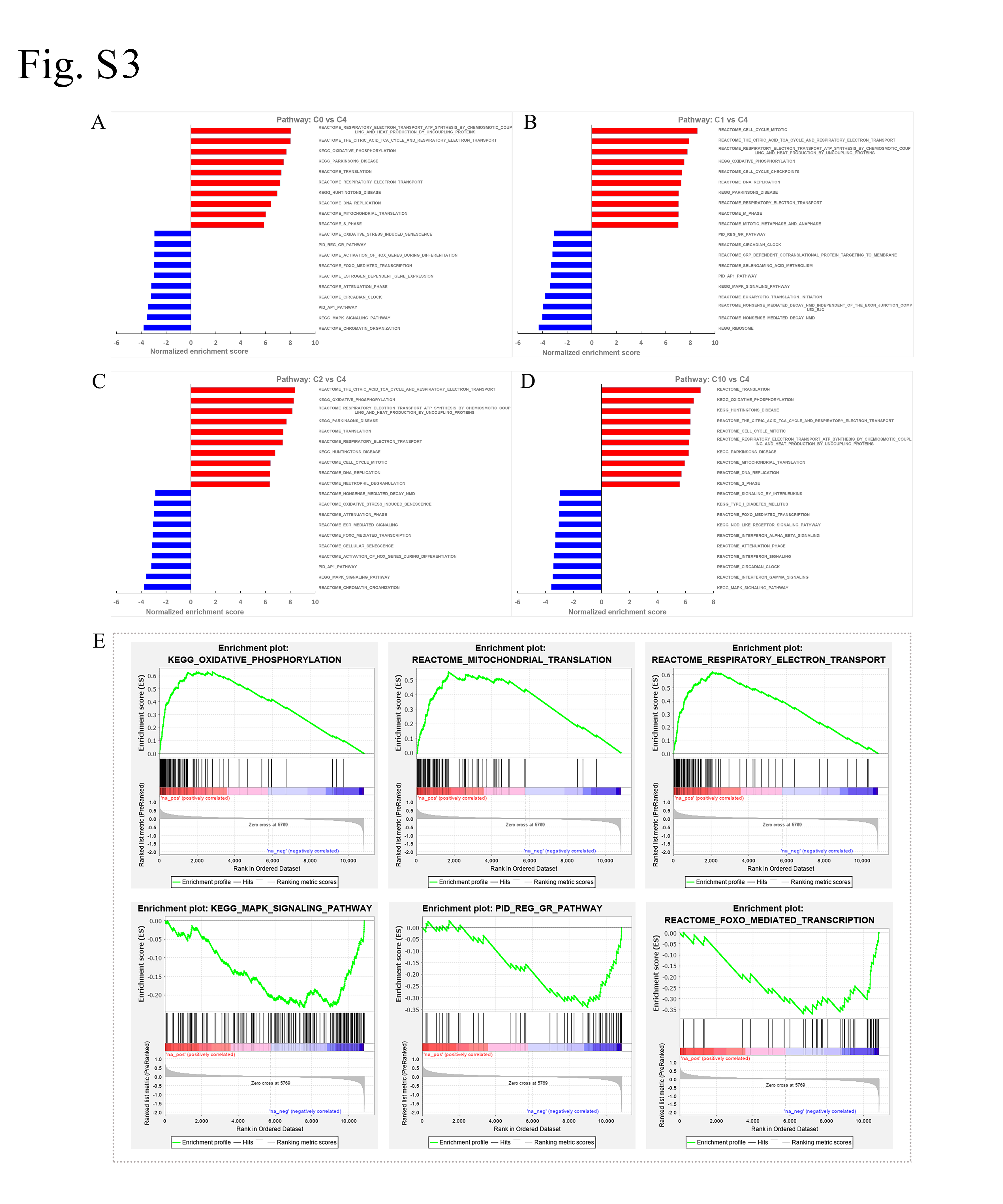

Supplement: Supplementary file 3 — SUPPORTING INFORMATION [file CTM2-11-e616-s011.tif]

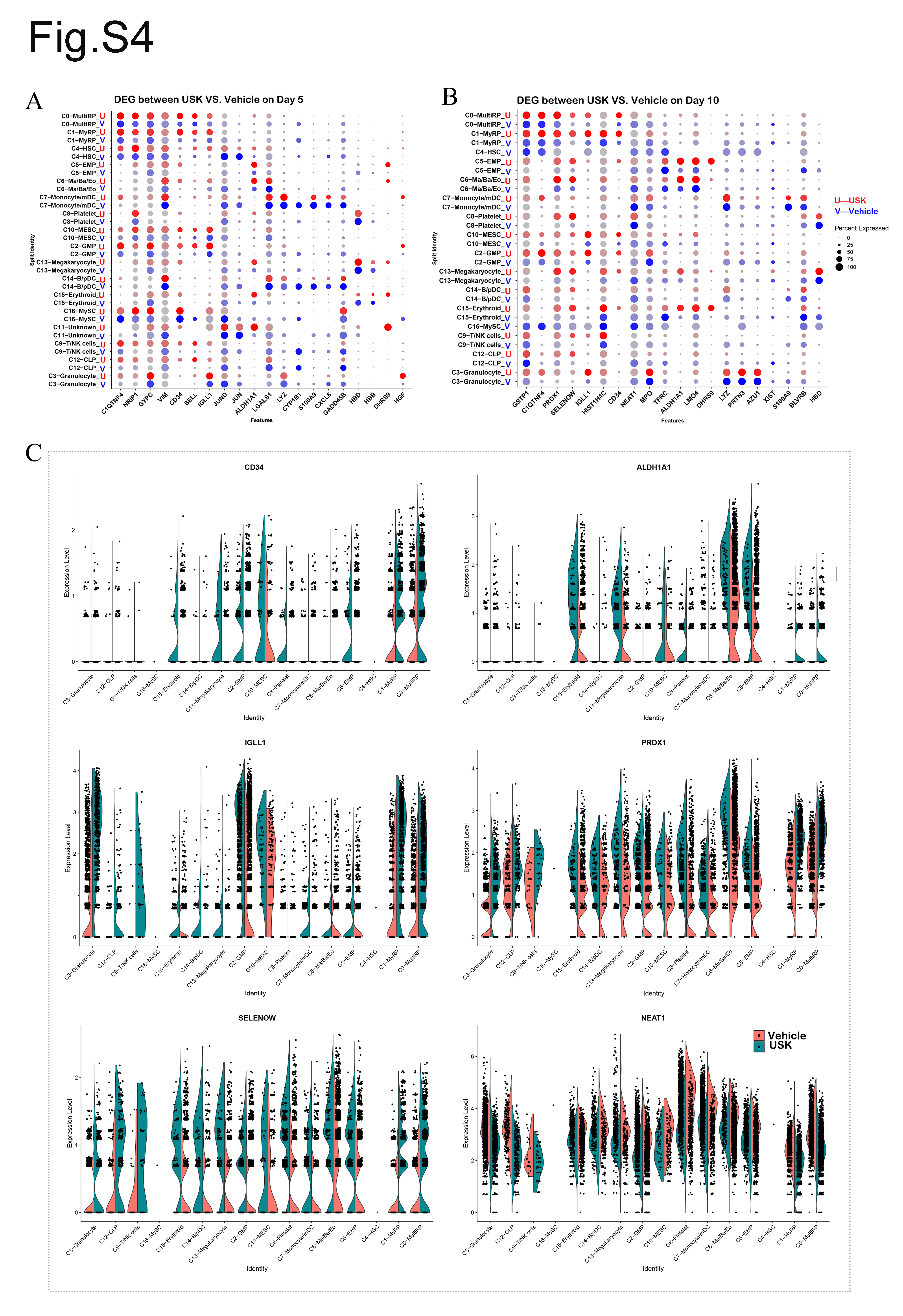

Supplement: Supplementary file 4 — SUPPORTING INFORMATION [file CTM2-11-e616-s014.tif]

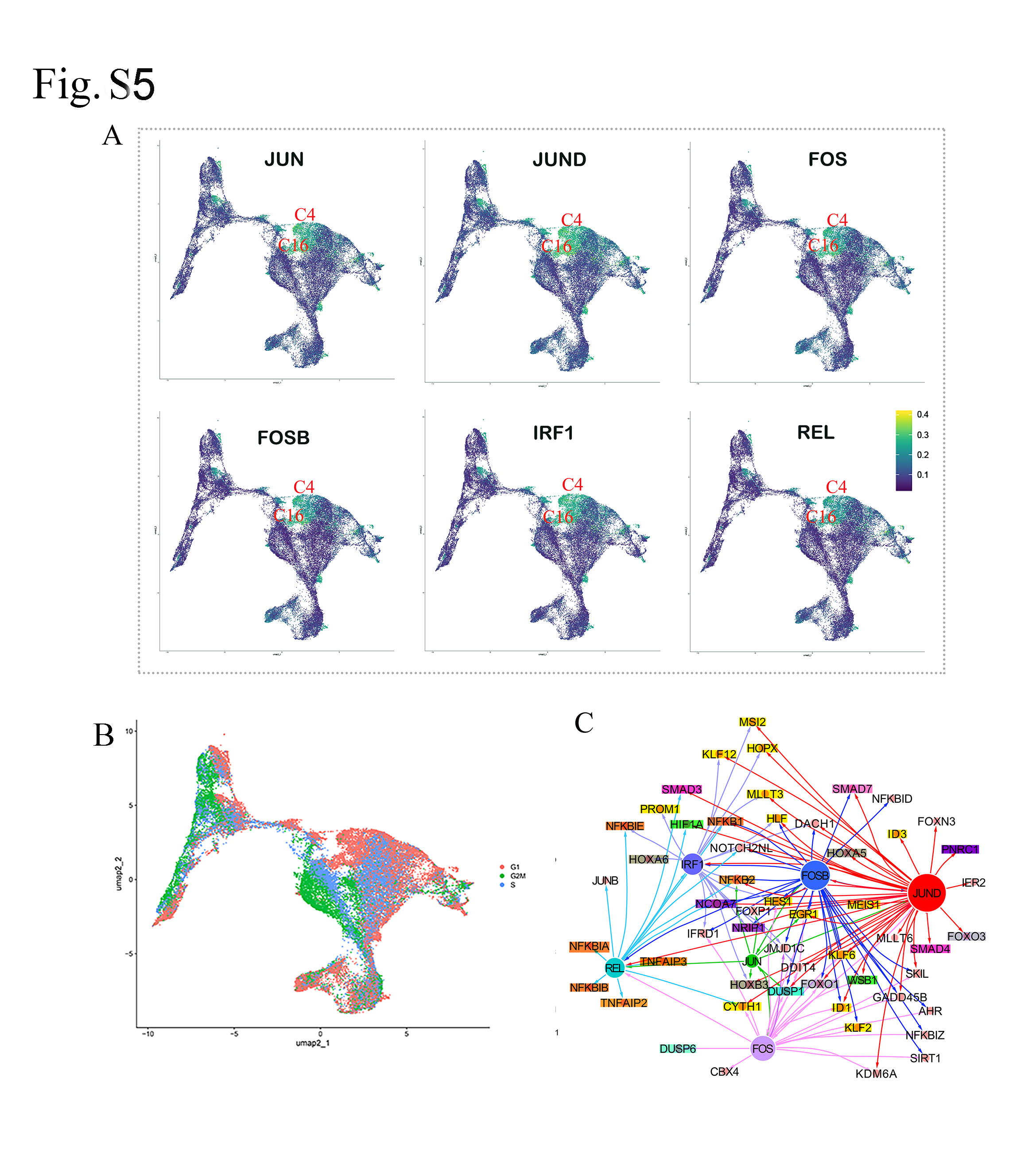

Supplement: Supplementary file 5 — SUPPORTING INFORMATION [file CTM2-11-e616-s008.tif]
