# Supplementary material for: Single‐cell RNA sequencing reveals the landscapes of human cord blood hematopoietic stem cell differentiation during ex vivo culture
Source: Clin Transl Med. 2021 Nov 8;11(11):e616. doi: 10.1002/ctm2.616 (PMC8574970; doi:10.1002/ctm2.616)
Supplement: Supplementary file 6 — SUPPORTING INFORMATION [file CTM2-11-e616-s004.docx]

|  | CD34^+^ cells  (Mean values ± SD)% | CD34^+^CD38^-^ cells  (Mean values ± SD)% | CD34^+^CD38^-^CD45RA^-^CD90^+^ cells (Mean values ± SD)% |
| --- | --- | --- | --- |
| Unculture | 93.74 ± 2.60 | 12.60 ± 6.79 | 1.25 ± 1.21 |
| Vehicle | 47.73 ± 7.78^#^ | 34.41 ± 4.82 | 0.52 ± 0.46 |
| UM171 | 73.27 ± 7.29^***#^ | 57.86 ± 12.46^***#^ | 2.15 ± 2.50 |
| SR1 | 73.75 ± 6.19^***#^ | 45.29 ± 9.51^#^ | 1.25 ± 1.02 |
| K1 | 66.21 ± 9.20^***#^ | 44.68 ± 8.11^#^ | 1.31 ± 1.21 |
| USK | 86.99 ± 8.92^***^ | 68.36 ± 13.91^***#^ | 5.38 ± 4.48^***#^ |

Supplementary table 1. Proportion of CD34^+^, CD34^+^ CD38^-^, and CD34^+^CD38^-^CD45RA^-^CD90^+^ cells (*n* = 11 independent experiments (n=11 independent experiments, Data shown as mean±SD, One Way ANOVA test). Note: Compared with Vehicle, *** Denotes *p* < 0.001; Compared with Unculture, ^#^*p* < 0.05.
