# Supplementary material for: Single‐cell RNA sequencing reveals the landscapes of human cord blood hematopoietic stem cell differentiation during ex vivo culture
Source: Clin Transl Med. 2021 Nov 8;11(11):e616. doi: 10.1002/ctm2.616 (PMC8574970; doi:10.1002/ctm2.616)
Supplement: Supplementary file 7 — SUPPORTING INFORMATION [file CTM2-11-e616-s013.docx]

|  | TNC | CD34^+^ cells | CD34^+^CD38^-^ cells | CD34^+^CD38^-^CD45RA^-^CD90^+^ cells |
| --- | --- | --- | --- | --- |
| Vehicle | 126.65 ± 34.42 | 64.03 ± 18.51 | 406.02 ± 227.19 | 61.58 ± 47.51 |
| UM171 | 135.96 ± 54.10 | 107.89 ± 49.88 | 765.18 ± 584.70^*^ | 248.56 ± 193.44 |
| SR1 | 153.82 ± 41.85 | 119.02 ± 24.06^***^ | 655.18 ± 380.58 | 210.05 ± 201.46 |
| K1 | 110.89 ± 35.31 | 76.55 ± 17.18 | 450.62 ± 213.59 | 133.08 ± 118.93 |
| USK | 107.02 ± 31.72 | 99.82 ± 33.33^*^ | 715.00 ± 411.34 | 543.33 ± 365.20^***^ |

Supplementary table 2. Expansion folds of TNC, CD34^+^, CD34^+^ CD38^-^, and CD34^+^CD38^-^CD45RA^-^CD90^+^ cells (*n*=11 independent experiments, Data shown as mean±SD, One Way ANOVA test). Note: Compared with vehicle group, *** Denotes *p* < 0.001; * Denotes *p* < 0.05. TNC, total nucleated cells.
