# Supplementary material for: Single‐cell RNA sequencing reveals the landscapes of human cord blood hematopoietic stem cell differentiation during ex vivo culture
Source: Clin Transl Med. 2021 Nov 8;11(11):e616. doi: 10.1002/ctm2.616 (PMC8574970; doi:10.1002/ctm2.616)
Supplement: Supplementary file 8 — SUPPORTING INFORMATION [file CTM2-11-e616-s006.docx]

|  | G | M | GM | E | GEMM |
| --- | --- | --- | --- | --- | --- |
| Unculture | 87.1 ± 49.18 | 9.83 ± 10.63 | 27.33 ± 16.37 | 78.83 ± 44.97 | 44.83 ± 18.04 |
| Vehicle | 8.50 ± 6.19 | 28.17 ± 14.8 | 20.50 ± 9.07 | 51.00 ± 28.91 | 1.83 ± 2.14^**^ |
| UM171 | 12.00 ± 12.59 | 37.67 ± 24.95 | 18.17 ± 14.65 | 97.83 ± 91.49 | 4.33 ± 4.50 |
| SR1 | 8.17 ± 9.99^*^ | 26.00 ± 12.17 | 14.17 ± 11.69 | 64.83 ± 43.70 | 2.00 ± 2.10^*^ |
| K1 | 11.67 ± 12.48 | 23.00 ± 9.88 | 19.67 ± 19.52 | 83.17 ± 54.63 | 4.00 ± 4.34 |
| USK | 12.83 ± 7.05 | 23.33 ± 13.92 | 14.67 ± 7.00 | 109.83 ± 137.31 | 4.83 ± 5.46 |

Supplementary Table 3. CFU numbers after 14 day in culture (n=6 independent experiments, Data shown as mean±SD, Kruskal-Wallis test). CFU, colony-forming units; G, granulocyte; M, macrophage; GM, granulocyte-macrophage; E, erythrocyte; GEMM, granulocyte/ erythrocyte/macrophage/ megakaryocyte. Note: Compared with Unculture group, ** Denotes *p* < 0.01;* Denotes *p* < 0.05.
