# Supplementary material for: Single‐cell RNA sequencing reveals the landscapes of human cord blood hematopoietic stem cell differentiation during ex vivo culture
Source: Clin Transl Med. 2021 Nov 8;11(11):e616. doi: 10.1002/ctm2.616 (PMC8574970; doi:10.1002/ctm2.616)
Supplement: Supplementary file 9 — SUPPORTING INFORMATION [file CTM2-11-e616-s015.docx]

| Proportion of hCD45^+^ cells in PB and BM of primary recipients of 500 starting dose | | | | | |
| --- | --- | --- | --- | --- | --- |
| Group | 5 w | 8 w | 12 w | 16 w (PB) | 16 w (BM) |
| Uncultured (n=7) | 0.15±0.08 | 0.84±0.58 | 3.67±5.72 | 1.56±1. 18 | 2.52±3.46 |
| Vehicle (n=6) | 0.21±0.08 | 0.73±0.73 | 1.33±0.37 | 0.40±0. 31^*^ | 0.03±0.03^*^ |
| USK (n=6) | 0.45±0.17^*#^ | 0.52±0.62 | 1.47±0. 85 | 0.90±0. 54 | 1.27±2.03^#^ |
| Proportion of hCD45^+^ cells in PB and BM of primary recipients of 2500 starting dose | | | | | |
| Group | 5 w | 8 w | 12 w | 16 w (PB) | 16 w (BM) |
| Uncultured (n=7) | 1.14±0.65 | 4.13±1.99 | 10.69±6.83 | 8.90±4.05 | 36.48±18.99 |
| Vehicle (n=6) | 0.38±0.15^*^ | 0.87±0.78^*^ | 1.57±0.65^*^ | 0.55±0. 24^*^ | 1.11±0.23^*^ |
| USK (n=6) | 0.40±0.21^*^ | 0.72±0.28^*^ | 0.92±0.61^*^ | 0.58±0.30^*^ | 1.21±1.25^*^ |
| Proportion of hCD45^+^ cells in PB and BM of primary recipients of 10000 starting dose | | | | | |
| Group | 5 w | 8 w | 12 w | 16 w (PB) | 16 w (BM) |
| Unculture (n=12) | 3.98±1. 54 | 13.33±8.81 | 15.33±7.28 | 11.18±7.21 | 42.93±14.19 |
| Vehicle (n=11) | 1.85±1.48^*^ | 6.01±6.42 | 9.07±4.54 | 5.64±3. 94 | 17.19±9.22^*^ |
| USK (n=11) | 3.35±5.13 | 3.92±4.37^*^ | 4.14±3.89^*#^ | 3.79±4.46^*^ | 27.65±13.92^*^ |

Supplementary Table 4. Proportion of hCD45^+^ cells in PB and BM of primary recipients (Data shown as mean±SD, Kruskal-Wallis test). Note: Compared with uncultured, * Denotes *p* < 0.05; Compared with Vehicle, # Denotes *p* < 0.05.
