# Supplementary material for: Single‐cell RNA sequencing reveals the landscapes of human cord blood hematopoietic stem cell differentiation during ex vivo culture
Source: Clin Transl Med. 2021 Nov 8;11(11):e616. doi: 10.1002/ctm2.616 (PMC8574970; doi:10.1002/ctm2.616)
Supplement: Supplementary file 10 — SUPPORTING INFORMATION [file CTM2-11-e616-s010.docx]

A

| Culture conditions | Cell number transplanted | | Number of mice with ≥0.2% human  cell chimerism/total number of mice |
| --- | --- | --- | --- |
| Unculture | 10000 | | 12/12 |
|  | 2500 | | 7/7 |
|  | 500 | | 4/7 |
|  | Equivalent  Starting Dose | Dose after Expansion |  |
| Vehicle | 10000 | 1.89×10^6^ | 11/11 |
|  | 2500 | 4.73×10^5^ | 6/6 |
|  | 500 | 9.45×10^4^ | 0/6 |
| USK | 10000 | 1.09×10^6^ | 11/11 |
|  | 2500 | 2.73×10^5^ | 6/6 |
|  | 500 | 5.46×10^4^ | 5/6 |

B

| Culture  conditions | SRC frequency  per starting cell | 95%Confidence  Interval | SRC numbers  per 10^6^ CD34^+^  starting cells | SRC frequency in  total number of  cells transplanted | 95% Confidence Interval |
| --- | --- | --- | --- | --- | --- |
| Unculture | 1/543 | 1/235 – 1/1260 | 1841 | 1/543 | 1/235 – 1/1260 |
| Vehicle | 1/1379 | 1/622 – 1/3056 | 725 | 1/260631 | 1/117558 – 1/577584 |
| USK | 1/279^*^ | 1/104 – 1/751 | 3584 | 1/30411 | 1/11336 – 1/81859 |

Supplementary Table 5. Limiting dilution analysis of primary NPG recipient engraftment.

(A) Summary of primary NPG recipient engraftment data. (B) Primary Limiting Dilution Analysis to determine the SRC frequency by ELDA software at 16 weeks post transplantation. Data from Supplementary Table 5A. (Chi-square test). Note: Compared with Vehicle, * Denotes p < 0.05.
