# Supplementary material for: Single‐cell RNA sequencing reveals the landscapes of human cord blood hematopoietic stem cell differentiation during ex vivo culture
Source: Clin Transl Med. 2021 Nov 8;11(11):e616. doi: 10.1002/ctm2.616 (PMC8574970; doi:10.1002/ctm2.616)
Supplement: Supplementary file 11 — SUPPORTING INFORMATION [file CTM2-11-e616-s007.docx]

A

| Culture conditions | Total number of BM  cells injected per  secondary recipient | Human BM cells  transplanted | Number of mice with ≥0.2% human  cell chimerism/total number of mice |
| --- | --- | --- | --- |
| Unculture | 1×10^7^ | 4293000 | 6/8 |
|  | 5×10^6^ | 2146500 | 0/6 |
|  | 2×10^6^ | 858600 | 3/6 |
| Vehicle | 1×10^7^ | 1719000 | 1/6 |
|  | 5×10^6^ | 859500 | 1/6 |
|  | 2×10^6^ | 343800 | 2/5 |
| USK | 1×10^7^ | 2765000 | 3/6 |
|  | 5×10^6^ | 1382500 | 0/6 |
|  | 2×10^6^ | 553000 | 1/6 |

B

| Culture conditions | SRC frequency per starting cell | 95% Confidence Interval |
| --- | --- | --- |
| Unculture | 1/3988287 | 1/2027133 – 1/7846764 |
| Vehicle | 1/3870533 | 1/1403170 – 1/10676557 |
| USK | 1/5876406 | 1/2205894 – 1/15654496 |

Supplementary Table 6. Limiting dilution analysis of secondary NPG recipient engraftment.

(A) Summary of secondary NPG recipient engraftment data. (B) Poisson statistics were applied to the data in A. SRC frequency in BM of secondary NPG recipients (Chi-square test).
