# Supplementary material for: Single‐cell RNA sequencing reveals the landscapes of human cord blood hematopoietic stem cell differentiation during ex vivo culture
Source: Clin Transl Med. 2021 Nov 8;11(11):e616. doi: 10.1002/ctm2.616 (PMC8574970; doi:10.1002/ctm2.616)
Supplement: Supplementary file 12 — SUPPORTING INFORMATION [file CTM2-11-e616-s016.docx]

| Group | Proportion of hCD45^+^ cells in PB | | | Proportion of hCD45^+^ cells in BM | | |
| --- | --- | --- | --- | --- | --- | --- |
|  | 2×10^6^ | 5×10^6^ | 1×10^7^ | 2×10^6^ | 5×10^6^ | 1×10^7^ |
| Unculture | 0.20 ± 0.20  (n=6) | 0.10±0.00  (n=6) | 0.34± 0.22  (n=8) | 0.31±0.33  (n=6) | 0.08±0.04  (n=6) | 0.74± 1.40  (n=8) |
| Vehicle | 0.30 ±0.14  (n=5) | 0.43 ± 0.37  (n=6) | 0.33± 0.27  (n=6) | 0.10±0.09  (n=5) | 0.27±0.49  (n=6) | 0.10 ± 0.13  (n=6) |
| USK | 0.37 ±0.22  (n=6) | 0.18±0.12  (n=6) | 0.82± 0.85  (n=6) | 0.14±0.12  (n=6) | 0.08±0.06  (n=6) | 0.18 ± 0.15  (n=6) |

Supplementary Table 7. Proportion of hCD45^+^ cells in PB and BM of secondary recipients at 16 weeks post-transplantation(n = 5-8 for each group, Data shown as mean±SD).
