# Supplementary material for: Single‐cell RNA sequencing reveals the landscapes of human cord blood hematopoietic stem cell differentiation during ex vivo culture
Source: Clin Transl Med. 2021 Nov 8;11(11):e616. doi: 10.1002/ctm2.616 (PMC8574970; doi:10.1002/ctm2.616)
Supplement: Supplementary file 14 — SUPPORTING INFORMATION [file CTM2-11-e616-s012.docx]

| Cluster | Unculture | Day 5 Vehicle | Day 5 USK | Day 10 Vehicle | Day 10 USK |
| --- | --- | --- | --- | --- | --- |
| C0-MultiRP | 11.98% | 15.75% | 22.08% | 10.14% | 13.99% |
| C1- MyRP | 3.10% | 17.58% | 19.89% | 6.8% | 12.52% |
| C2- GMP | 4.45% | 16.76% | 17.62% | 15.28% | 16.20% |
| C3- Granulocyte | 3.47% | 7.09% | 5.36% | 20.88% | 6.54% |
| C4- HSC | 36.83% | 0.13% | 0.12% | 0.01% | 0.00% |
| C5- EMP | 4.35% | 6.72% | 7.42% | 4.78% | 9.28% |
| C6- Ma/Ba/Eo | 2.99% | 3.20% | 4.35% | 2.64% | 17.98% |
| C7- Monocyte/mDC | 1.99% | 6.74% | 3.10% | 11.99% | 4.13% |
| C8- Platelet | 1.73% | 5.39% | 2.40% | 12.23% | 3.81% |
| C9- T/NK | 1.89% | 1.54% | 1.21% | 0.26% | 0.43% |
| C10- MESC | 7.54% | 2.81% | 3.70% | 0.83% | 1.40% |
| C11- Unknown | 5.13% | 0.03% | 0.02% | 0.00% | 0.00% |
| C12- CLP | 4.86% | 1.52% | 1.96% | 2.29% | 2.54% |
| C13-Megakaryocyte | 1.15% | 3.11% | 1.47% | 4.10% | 1.97% |
| C14- B/pDC | 1.52% | 2.37% | 1.96% | 2.73% | 1.91% |
| C15- Erythroid | 1.53% | 9.24% | 7.32% | 5.04% | 7.29% |
| C16- MySC | 5.51% | 0.03% | 0.01% | 0.01% | 0.00% |
| Total | 100% | 100% | 100% | 100% | 100% |

Supplementary Table 9. Proportion of cell subpopulation under different culture conditions.
