# Supplementary material for: Single‐cell RNA sequencing reveals the landscapes of human cord blood hematopoietic stem cell differentiation during ex vivo culture
Source: Clin Transl Med. 2021 Nov 8;11(11):e616. doi: 10.1002/ctm2.616 (PMC8574970; doi:10.1002/ctm2.616)
Supplement: Supplementary file 15 — SUPPORTING INFORMATION [file CTM2-11-e616-s001.docx]

**Supplemental Figure 1.** scRNA-seq show that HSCs in CB CD34^+^ cells have undergone differentiation after *ex vivo* culture. (A) Heatmaps showing the expression of top 5 enriched genes in every cluster. Expression values are scaled (z-scored) for visualization. (B) Representative marker genes projected on UMAP maps. The colour gradient indicates levels of expression. (C) Bar chart of cell subpopulation proportion under different culture conditions.

**Supplemental Figure 2.** Identification of key molecules regulating HSC self-renewal and differentiation. (A) Dot plot showing differential expression genes (DEG) between day 5 USK and Unculture in the defined cell types. (B) Dot plot showing differential expression genes (DEG) between day 10 USK and Unculture in the defined cell types. Size of the dot is the percentage of cells expressing this gene in each group. (C) Heatmaps showing the differential expression genes (DEG) between cluster 0, 1, 2, 10, 16 and cluster 4.

**Supplemental Figure 3.** Pathway enrichment analysis of differentially expressed genes (DEG) in cluster 0, 1, 2, 10 versus cluster 4. (A) Cluster 0 versus cluster 4. (B) Cluster1 versus cluster 4. (C) Cluster 2 versus cluster 4. (D)Cluster 10 versus cluster 4. (E) Enrichment plot of the gene sets.

**Supplemental Figure 4.** Differential expression genes (DEG) between USK and Vehicle expanded cells. (A) Dot plot showing differential expression genes (DEG) between USK and Vehicle on day 5 in the defined cell types. (B) Dot plot showing differential expression genes(DEG) between USK and Vehicle on day 10 in the defined cell types. Size of the dot is the percentage of cells expressing this gene in each group. (C) Violin plots showing the expression of representative DEG between USK and Vehicle groups on day 10.

**Supplemental Figure 5.** SCENIC analysis from CB CD34^+^ cells scRNA-seq data. (A) Regulatory intensity of HSC related transcription factors (JUN, JUND, FOS, FOSB, IRF1, REL) projected on UMAP maps. (B) UMAP plot showing the cell cycle status of each cell. (C) Predicted network for HSC related transcription factors (JUN, JUND, FOS, FOSB, IRF1, REL) target genes.
