# Supplementary material for: Single‐cell RNA sequencing reveals the landscapes of human cord blood hematopoietic stem cell differentiation during ex vivo culture
Source: Clin Transl Med. 2021 Nov 8;11(11):e616. doi: 10.1002/ctm2.616 (PMC8574970; doi:10.1002/ctm2.616)
Supplement: Supplementary file 16 — SUPPORTING INFORMATION [file CTM2-11-e616-s009.doc]

**MATERIALS AND METHODS**

**1. Ethics statement and cord blood sample collection**

All experiments in this study were performed in accordance with the approved protocol by the Ethics Committee of the Fifth Medical Center of Chinese PLA General Hospital (ky-2018-5-38). Informed consent for the collection of CB samples was obtained.

**2. Mice**

NPG mice (NOD-Prkdc^scid^Il2rg^null^/Vst, stock No. VS-AM-001) were obtained from Beijing Vitalstar Biotechnology Co., Ltd. Animal experiments were conducted using protocols that have been approved by the IACUC of Beijing Vitalstar Biotechnology Co., Ltd.

**3. CB** **CD34^+^ cells isolation, culture and grouping**

CD34^+^ cells from fresh CB were isolated as our previously described[1] and were cultured in serum-free StemSpanSFEM Ⅱ (StemCell Technologies, Canada) supplemented with the cytokines 100 ng/ml FMS-like trypsin kinase 3 ligand (Ftl3L), human stem cell factor (SCF), IL-6 and thrombopoietin (TPO) (PeproTech, USA). The CD34^+^ cells were divided into five groups: vehicle (0.1%DMSO; Sigma-Aldrich), SR1 (1 μM; Selleck Chemicals), UM171 (35 nM; Selleck Chemicals), JNK-IN-8 (K1; 2 μM; Gifted by Professor Hongkui Deng from Peking University), and USK (UM171+SR1+K1). CD34^+^ cells (5 × 10^4^ cells/2 mL) were cultured in six-well plates at 37 °C under 5% CO_2_. The culture medium was refreshed on day 5 and the cytokines and small molecules in the respective groups were supplemented. Cultures were harvested on day 10.

**4. Flow cytometry**

The following anti-human antibodies (BioLegend) was used to detect cell phenotypes: CD34-PE, CD38-Brilliant Violet 510 or CD38-APC, CD45RA-APC-Cy7, CD90-FITC, CD33-PE, CD3-PE/Dazzle 594, CD19-APC, CD235a-PE/Cy7, CD41-Brilliant Violet 510 and CD56-Brilliant Violet 421. Briefly, cells were incubated with the corresponding antibodies at 4 °C for 30 min, washed with phosphate-buffered saline (GIBCO), and then fixed in 4% formaldehyde buffer before flow cytometry (Beckman Coulter).

**5. Colony-forming unit assay**

Cells (1 × 10^3^ / dish) were seeded into cytokine-containing methylcellulose media (MethoCult MediaH4435, StemCell Technologies) and then cultured for 14 days to generate colony-forming units (CFUs). Multilineage colonies including CFU granulocytes (CFU-G); CFU macrophages (CFU-M); burst-forming units-erythroid (BFU-E); CFU granulocyte-macrophage (CFU-GM), CFU erythrocytes (CFU-E); and CFU-granulocytes, erythrocytes, macrophages, and megakaryocytes (CFU-GEMM), were visualized and counted under an inverted microscope (Nikon).

**6. Primary** **transplantation**

Six-week-old female NPG mice were irradiated with 160 cGy. Transplantation of freshly isolated CD34^+^ CB cells or their progeny in 10-day cultures (1 × 10^4^ cells) by tail vein injection was performed within 24 h after irradiation. The percentage of human CD45^+^ cells in peripheral blood at 5, 8, 12, and 16 weeks posttransplantation was detected by flow cytometry. Engraftment and hematopoietic reconstitution of multiple lineages in the bone marrow of primary recipient mice were monitored by flow cytometry at week 16.

Engraftment and hematopoietic reconstitution were detected using the following antibodies (BioLegend): anti-human CD45-FITC, CD19-APC, CD33-PE, CD235a-PE/Cy7, CD3-PE/Dazzle594, CD41-Brilliant Violet 510, and CD56-Brilliant Violet 421; and anti-mouse CD45.1-APC/Cy7.

**7. Secondary transplantation**

Six-week-old female NPG mice secondary transplant recipients were irradiated with 160 cGy. Serial transplantation of bone marrow cells (2×10^6^, 5×10^6^, and 1×10^7^) from primary recipient mice was performed within 24 h after irradiation. Bone marrow cells from secondary NPG mice were collected by flushing the femurs and tibias at 16 weeks posttransplantation. The proportion of human CD45^+^ cells in the bone marrow at 16 weeks posttransplantation was detected by flow cytometry.

1. **Limiting dilution assay**

Limiting dilution assay (LDA) was performed to quantify the frequency of hematopoietic human cells present in the severe-combined immunodeficient NPG mice serial transplant recipients (SRCs) as previously described [2](<http://bioinf.wehi.edu.au/software/elda/index.html>). The data was analyzed using the chi-square and Mann-Whitney U tests.

**9. scRNA-seq library preparation and sequencing**

Single-cell suspensions were loaded into a 10X Chromium controller and converted to barcoded scRNA-seq libraries to capture 1×10^4^ cells using the Chromium single-cell 3’ kit (V3 chemistry), according to the standard protocol. Library preparation and sequencing were performed according to the manufacturer’s standard protocol. Sequencing (150-nucleotides paired-end) of the single-cell libraries was performed using the Illumina NovaSeq 6000 instrument.

**10. scRNA-seq data processing**

Raw reads were processed using the Cell Ranger Single-Cell Software Suite (version 3.1.0, 10X Genomics Inc.). Briefly, the CellRanger *mkfastq* command was used to generate the demultiplexed FASTQ files (150 bp paired-end). The CellRanger *count* command was used for primary data analyses, which included filtering, alignment, UMI (unique molecular identifier) count quantification and barcode counting to generate a gene-barcode matrix, clustering, quality control and statistical analysis. Ensembl build 93 was used to annotate gene positions followed by filtering for biotype (only protein-coding, long intergenic noncoding RNA, antisense, immunoglobulin or T-cell receptor).

**11. Single-cell gene expression quantification and determination of major cell types**

We used CellRanger to generate raw gene expression matrices for each sample before importing them into the R version 3.6.0 software. The matrices were then converted to a Seurat object using the Seurat R package (version 3.1.0)[3]. Dead cells and doublets were removed as follows: 1) count the total number of UMIs and genes and the percentage of UMIs derived from the mitochondrial genome for each cell; 2) calculate the upper and lower limits (mean ± 2 standard deviations [SD]) for both the total UMIs and genes; 3) discard cells that had over 15% UMIs derived from mitochondrial genome; and 4) remove cells with total UMI or gene numbers outside of the upper and lower limits. For the remaining 43,463 out of 47,548 cells, gene expression matrices were normalized such that the number of UMIs in each was equal to 10,000 and log transformed The Seurat *SCTransform* function was used to select highly variable genes (HVGs) from the normalized data, and the top 2000 HVGs were used for clustering analysis and dimensionality reduction. The Seurat *RunPCA* function was performed to select the principal components (PCs) with the highly significant differences, which were subsequently plotted using the *RunUMAP* function (parameter “dim=1:25”). Batch effect correction was then performed using Harmony[4]. The first 30 “harmony” aligned coordinates were plotted using the *FindNeighbors* function (parameter “dim=1:30”). Cells were clustered into different groups using the *FindClusters* function (parameter “*resolution = 0.15*”). Cell clusters were assigned to known biological cell types based on canonical marker genes. Trajectory and pseudotime analysis were performed using Monocle 3[5].Cell cycle analysis was performed by using the Seurat program.

**12. Identification of marker genes and differentially expressed genes (DEGs)**

We used the Seurat *FindMarkers* function (default parameter of the “MAST” test) to identify marker genes and DEGs for the determined major cell types. We compared the gene expression values of cells from the clusters of interest to those of cells from the remaining clusters to identify marker genes based on the following criteria: 1) have an average expression value in the cluster of interest with at least 1.2-fold higher than the average expression in the remaining clusters; 2) have the highest mean expression in the cluster of interest compared to the rest of clusters; and 3) have more than 10% of detectable cells in the cluster of interest. We compared the gene expression values of cells between two paired treatments for each cluster of interest to identify DEGs for each cell type based on the same criteria applied to identify marker genes. Marker gene lists and whole DEG lists without any filtering were used as input for gene-set enrichment analysis (GSEA). **13. Gene function and pathway analysis**

Pathway enrichment was performed by GSEA version 4.03. Gene Ontology (GO) term analysis was performed using the Molecular Signatures Database (MSigDB, C2 and C5, Version 7.01)[6].

**14. Mapping cell-type-specific regulatory networks using SCENIC**

The 3-step single-cell regulatory network inference and clustering (SCENIC) workflow was applied to the datasets after clustering of the cells and cell type annotation[7]. Firstly, coexpression modules between candidate target genes and TFs were inferred using GENIE3. Next, modules with significantly enriched regulator's binding motif across target genes were identified by RcisTarget and regulons with only direct targets were created. In the last step of the workflow, the activity of each regulon in each cell was scored by AUCell to output a binarized activity matrix. Cell states were predicted based on the shared activity of regulatory subnetworks. Finally, a two-dimensional UMAP plot of the regulon-by-cell activity matrix that represent the cell states was generated.

**15. Statistical analysis**

Data following a normal distribution are presented as the mean ± SD unless otherwise indicated. Statistical analyses were performed using the SPSS v20 software (IBM, USA). Comparisons among groups were performed using one-way ANOVA, Kruskal-Wallis test or Mann-Whitney U tests. Values of *P* < 0.05 were considered statistically significant.

**References**

1 Wen R, Dong C, Xu C et al. UM171 promotes expansion of autologous peripheral blood hematopoietic stem cells from poorly mobilizing lymphoma patients [in eng]. International immunopharmacology 2020;81:106266.

2 Hu Y, Smyth GK. ELDA: extreme limiting dilution analysis for comparing depleted and enriched populations in stem cell and other assays [in eng]. J Immunol Methods 2009;347(1-2):70-78.

3 Butler A, Hoffman P, Smibert P et al. Integrating single-cell transcriptomic data across different conditions, technologies, and species [in eng]. Nat Biotechnol 2018;36(5):411-420.

4 Korsunsky I, Millard N, Fan J et al. Fast, sensitive and accurate integration of single-cell data with Harmony [in eng]. Nat Methods 2019;16(12):1289-1296.

5 Qiu X, Mao Q, Tang Y et al. Reversed graph embedding resolves complex single-cell trajectories [in eng]. Nat Methods 2017;14(10):979-982.

6 Subramanian A, Tamayo P, Mootha VK et al. Gene set enrichment analysis: a knowledge-based approach for interpreting genome-wide expression profiles [in eng]. Proceedings of the National Academy of Sciences of the United States of America 2005;102(43):15545-15550.

7 Aibar S, González-Blas CB, Moerman T et al. SCENIC: single-cell regulatory network inference and clustering [in eng]. Nat Methods 2017;14(11):1083-1086.
